# Risk of Severe Illness and Risk Factors of Outcomes of COVID-19 in Hospitalized Patients with Chronic Liver Disease in a Major U. S. Hospital Network

**DOI:** 10.1155/2022/8407990

**Published:** 2022-11-03

**Authors:** Arunkumar Krishnan, Laura Prichett, Yisi Liu, Peng-sheng Ting, Saleh A. Alqahtani, Amy K. Kim, Michelle Ma, James P. Hamilton, Tinsay A. Woreta, Po-Hung Chen

**Affiliations:** ^1^Division of Gastroenterology and Hepatology, Johns Hopkins University School of Medicine, Baltimore, MD 21287, USA; ^2^Department of Pediatrics, Johns Hopkins University School of Medicine, Baltimore, MD 21287, USA; ^3^Liver Transplant Center, King Faisal Specialist Hospital and Research Center, Riyadh 12713, Saudi Arabia

## Abstract

**Methods:**

We studied 2731 patients with known CLD who were hospitalized at the Johns Hopkins Health System with COVID-19 between March 1, 2020, and December 15, 2021. The primary outcome was all-cause mortality, and secondary outcomes were MV and vasopressors. Multivariable Cox regression models were performed to explore factors associated with the outcomes.

**Results:**

Overall, 80.1% had severe COVID-19, all-cause mortality was 8.9%, 12.8% required MV, and 11.2% received vasopressor support. Older patients with underlying comorbidities were more likely to have severe COVID-19. There was association between elevated aminotransferases and total bilirubin with more severe COVID-19. Hepatic decompensation was independently associated with all-cause mortality (HR 2.94; 95% CI 1.23–7.06). Alcohol-related liver disease (ALD, HR 2.79, 95% CI, 1.00–8.02) was independently associated with increased risk for MV, and independent factors related to vasopressor support were chronic pulmonary disease and underlying malignancy.

**Conclusions:**

COVID-19 infection in patients with CLD is associated with poor outcomes. SARS-CoV-2 infection in patients with hepatic decompensation was associated with an increased risk of in-hospital mortality hazard, and ALD among patients with COVID-19 was associated with an increased hazard for MV.

## 1. Introduction

Coronavirus disease 2019 (COVID-19) has been a devastating infectious disease, with a rapid surge in cases and deaths since first documented in Wuhan, China, in December 2019 [1]. As of September 7, 2022, its causative agent, the severe acute respiratory syndrome coronavirus 2 (SARS-CoV-2), is responsible for over 603 million confirmed cases and nearly 6.4 million deaths globally [2]. The clinical severity of COVID-19 varies from asymptomatic to fatal [3]. Available data have shown that known risk factors associated with poor outcomes in patients with COVID-19 include older age and underlying comorbidities such as obesity, hypertension, diabetes, chronic liver disease (CLD), and heart disease [4, 5]. A meta-analysis including 51,225 patients showed a pooled OR of 1.09 for obesity (95% CI: 0.84 to 1.41), 2.12 for diabetes (95% CI: 1.79 to 2.52), 2.61 for hypertension (95% CI: 2.19 to 3.17), 2.98 for cardiovascular disease (95% CI: 2.51 to 3.53) and 1.80 for CLD (95% CI: 1.35 to 2.39)^.^ [6] However, it remains unclear to what extent CLD should be considered a risk factor due to a shortage of appropriate studies [7]. Additionally, the global burden of CLD is vast and has been steadily increasing over the years. Ominously, CLD causes approximately 2 million deaths per year worldwide [8].

Available data suggest that patients with CLD who acquire COVID-19 have high hospitalization rates, and the mortality risk is close to 30–40% [9–11]. Still, preliminary studies were limited, and it remains unknown whether all patients with CLD or particular subgroups are at an increased risk for COVID-19-related adverse outcomes. First, existing data are controversial on the outcome following COVID-19 infection in patients with CLD and making it difficult to determine a prognosis for these patients [9, 12, 13]. Moreover, earlier studies collected data during the early pandemic when variants were not prevalent. An extensive, granular, representative clinical study is required to improve our understanding of the risk factors and severity of COVID-19 among patients with CLD. Finally, there is an under-representation of data about whether patients with CLD have an increased risk of the most intensive care of vasopressor support or mechanical ventilation. To address the abovementioned knowledge gaps, we analyzed a cohort of hospitalized patients with CLD and COVID-19 from a large health system in the United States. Our analysis focused on the independent associations between abnormal liver chemistry, clinical severity, and the risk of in-hospital mortality.

## 2. Patients and Methods

### 2.1. Study Design and Setting

In our retrospective cohort study, we included consecutive adult patients (≥18 years of age) with laboratory-confirmed COVID-19 and the presence of pre-existing CLD (according to predefined International Classification of Diseases [ICD]-10 codes) (Supplementary Table 1) admitted from March 1, 2020, to December 15, 2021. We obtained data from JH-CROWN, the COVID-19 Precision Medicine Analytics Platform Registry. JH-CROWN is our institutional registry of patients with suspected or confirmed COVID-19. The registry obtains data on demographics, clinical characteristics, laboratory tests, treatments, and outcomes from the medical records. In the supplementary materials, we detailed our study's clinical classifications and definitions implemented for abnormal liver chemistries. The JH-CROWN data for our study was approved by the Johns Hopkins Institutional Review Board (IRB00246683).

### 2.2. Study Outcomes

The primary outcome was all-cause mortality after the index date of positive SARS-CoV-2 infection. Death was assessed at the end of the study period. Secondary outcomes were the need for vasopressor drugs or mechanical ventilation after the index date of infection. Clinical outcomes were observed until December 15, 2021, the final follow-up date.

### 2.3. Statistical Analysis

Categorical variables were compared using chi-squared tests and summarized as frequencies (percentages). Continuous variables were assessed with the Mann-Whitney-Wilcoxon test and presented as median (interquartile range [IQR]). Patients were considered right-censored if they were discharged from the hospital alive or remained in the hospital at the end of the follow-up. We assessed the time-to-event in days from the date of hospital admission to the date of in-hospital death or hospital discharge alive or end of follow-up. The event-free survival probability was calculated using the Kaplan–Meier method, where the log-rank test compared different groups for significance. Cox regression analysis was used to explore the factors associated with mortality, mechanical ventilation, or vasopressor support using hazard ratios (HR) and 95% confidence intervals (CI). Univariable analyses first identified potential risk factors associated with the risk of death, mechanical ventilation, or vasopressor support. Subsequently, age, gender, ethnicity, race, smoking status, body mass index, etiology of liver diseases, and all pre-existing comorbidities were adjusted in multivariable Cox proportional hazards models. We also used Cox models to estimate HRs for the grade of liver chemistry elevation associated with major outcomes. All tests were two-tailed and used a significance level of *P* values <0.05. Analyses were performed using Stata (version SE16; StataCorp, College Station, TX).

## 3. Results

### 3.1. Demographic and Clinical Characteristics

**Figure 1:**
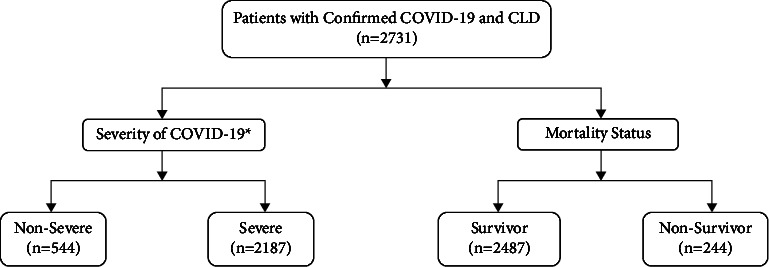
Flow chart of the study. ^∗^Severity of the diseases was based on the World Health Organization (WHO) interim guidance. COVID-19, coronavirus disease 2019; CLD, chronic liver disease.

Our study included 2731 hospitalized patients with CLD and confirmed SARS-CoV-2 infection (Figure 1). The median age of the study population was 61.3 (IQR, 45.4–74.1) years; 51.4% were female, and 34.3% were white (Table 1). The etiology of CLD was viral hepatitis (17.6%), nonalcoholic fatty liver disease (NAFLD) (5.4%), alcohol-related liver disease (ALD) (1.3%), and other types of liver diseases (93.3%). The majority (89.2%) of patients had noncirrhotic stage disease. Cirrhosis was well-compensated in only 28 (1.0%) patients at the time of analysis, and 267 (9.8%) patients had decompensated cirrhosis before diagnosis with COVID-19. The most common comorbidities were hypertension (HT) (89.3%), diabetes mellitus (DM) (63.3%), and chronic obstructive pulmonary disease (COPD) (27.4%).

### 3.2. Severe Disease

In all, 2187 (80.1%) patients were classified as severe cases during hospitalization. Patients in the severe group were older, obese, and likely to have more underlying comorbidities (Table 1). In addition, the severe disease was associated with significantly higher white blood cells, neutrophils, creatine, blood urea nitrogen (BUN), prothrombin time (PT), and international normalization ratio (INR). In contrast, absolute lymphocyte, albumin, and total protein levels were lower in patients with severe disease (Supplementary Table 2).

### 3.3. Analysis and Distribution of Abnormal Liver Chemistries during Hospitalization

Abnormal liver chemistries were more common in patients with severe disease; most patients had mild elevations within 1-2 × ULN (Table 1). The median alanine aminotransferase (ALT) and aspartate aminotransferase (AST) levels were 24 and 28 U/L, respectively, in nonsevere diseases compared to 30 and 38 U/L in severe cases (*P* < 0.001). Elevated aminotransferase levels at >2–5 × ULN and >5 × ULN were significantly more common among patients with severe diseases than nonsevere. The median level of alkaline phosphatase (ALP) was higher in patients with severe disease, whereas no difference was noted in median total bilirubin (T. Bil) between these two severities.

### 3.4. In-Hospital Management

The most frequently used specific therapies for COVID-19 included dexamethasone (37.3%), azithromycin (25.5%), and hydroxychloroquine (11.8%) (Supplementary Table 2). Patients with severe diseases had a significantly extended hospital stay than the nonsevere group (median 6.4 vs. 2.7 days, *P*  <  0.001).

### 3.5. Major Outcomes

#### 3.5.1. Risk Factors Associated with Mortality

Death occurred in 244 (8.9%) of the total CLD cohort; all belonged to the severe diseases group. Overall, 2412 (97.0%) were discharged at the time of data collection for this analysis. Furthermore, compared to survivors, nonsurvivors were older (52.4 vs. 62.6 years, *P* < 0.001) and had significantly more comorbidities (Table 1). Among patients with CLD, factors associated with all-cause mortality were increasing age (HR 1.05 per year; 95% CI 1.04–1.07) and hepatic decompensation (HR 2.94; 95% CI 1.23–7.06). In addition, abnormal INR, PT, CRP, and D-dimer were independently associated with mortality (Table 2). Among liver chemistries, elevated ALP (HR 1.02; 95% CI 1.00–1.03) and T. Bil (HR 1.25; 95% CI 1.15–1.37) were associated with the highest risk of in-hospital mortality. Compared to patients with normal-range AST, all-cause mortality risk significantly increased 1.92-fold (95% CI, 1.10–3.34) when AST >2–5 × ULN and 3.62-fold (95% CI, 1.52–8.64) when AST >5 × ULN. Patients with an elevated level of ALT >2–5 × ULN (HR 1.47; 95% CI, 1.29–2.77) were independently associated with higher mortality hazards than patients with ALT at the normal level. Likewise, compared to the patients with normal ALP, all-cause mortality risk significantly increased 1.68-fold (95% CI, 1.12–2.52) when ALP >1-2 × ULN and 2.29-fold (95% CI, 1.10–5.27) when ALP >2–5 × ULN. Viral hepatitis (HR 0.22; 95% CI, 0.09–0.50) and NAFLD (HR 0.21; 95% CI, 0.07–0.65) were associated with lower mortality hazards in multivariable analysis.

#### 3.5.2. Risk Factors Associated with Vasopressor Support and Mechanical Ventilation

In all, 307 (14%) patients with severe COVID-19 required vasopressors, and 350 (12.8%) received invasive mechanical ventilation. Patients who required vasopressor support or mechanical ventilation were older and more likely to have pre-existing comorbidities (Supplementary Table 3). In general, ALT, AST, and T. Bil values were significantly higher in these patients. However, there was no difference in median ALP in either of these outcomes. Furthermore, patients who required vasopressor support or mechanical ventilation had varying degrees of abnormal liver chemistries. COPD and underlying malignancy were associated with vasopressor support (Table 2). In addition, compared to patients with normal AST, the risk for vasopressors increased 1.32-fold (95% CI 1.00–1.76) when AST >1-2 × ULN and 2.81-fold (95% CI 1.18–6.71) when AST >5 × ULN. Abnormal ALT and T-Bil levels were not independently associated with an increased risk of vasopressor support. Viral hepatitis (HR 0.56, 95% CI, 0.37–0.85) was associated with lower vasopressor support hazards in multivariable analysis.

Factors significantly associated with mechanical ventilation included alcohol-related liver disease (ALD, HR 2.79, 95% CI, 1.00–8.02). Besides, compared to patients with normal AST, the risk for mechanical ventilation significantly increased 1.69-fold (95% CI 1.09–2.61) when AST >2–5 × ULN and 2.50-fold (95% CI 1.12–5.58) when AST >5 × ULN (Table 2). Viral hepatitis (HR 0.49; 95% CI 0.32–0.74) and NAFLD (HR 0.54; 95% CI 0.31–0.93) were associated with lower mechanical ventilation hazards in multivariable analysis.

## 4. Discussion

Our study has highlighted several important findings amongst a large cohort of CLD patients hospitalized with COVID-19 infection at a large academic center in the United States. First, we observed all-cause mortality of 8.9%; 12.8% required mechanical ventilation; and 11.2% received vasopressor support. Second, patients with decompensated cirrhosis were independently associated with an increased risk of COVID-19-related mortality. Additionally, baseline factors such as older age, high levels of T-Bil, and increased inflammatory markers such as CRP and D-Dimer were associated with death. Third, during hospitalization, a liver-specific factor associated with the need for mechanical ventilation from COVID-19 was ALD. In addition, we noticed that high serum neutrophil and BUN levels were independently associated with respiratory support through intubation. Fourth, we observed independent factors related to vasopressor support: chronic pulmonary disease and underlying malignancy. Fifth, there were strong associations between elevated AST or T. Bil with more severe COVID-19 infections.

In our study, hepatic decompensation was independently associated with a higher risk of COVID-19-related in-hospital mortality. Ge et al. have reported data from the N3C Consortium in the USA with more than 220,000 CLD patients highlighted the adverse impact of advanced liver disease, with cirrhosis being associated with a 2.38 times mortality hazard in an adjusted model of mortality 30-days following SARS-CoV-2 infection [14]. In addition, when compared with SARS-CoV-2 patients with cirrhosis/negative and patients with decompensated cirrhosis, SARS-CoV-2 positivity (cirrhosis/positive) was associated with a 2.20 times adjusted hazard of death within 30 days [12]. Similarly, Mallet et al. have reported the outcomes of hospitalized COVID-19 from the French National Hospital Discharge database with a cohort of >259,000 inpatients with COVID-19, including 15,476 with pre-existing CLD, and demonstrated that patients with decompensated cirrhosis were at 2.21 adjusted odds of increased adjusted risk for mortality, highlighting the importance of delineating cirrhosis severity when prognosticating outcomes [15]. In contrast to these findings, one nationwide Swedish CLD cohort did not demonstrate any associations between cirrhosis and COVID-19-related mortality [16]. However, this study only included patients with biopsy-proven CLD diagnoses prior to 2017. Therefore, more advanced liver disease may be under-represented because these patients were not subjected to biopsy or died before the onset of the pandemic. Patients with hepatic decompensation had a 2.9-fold increased mortality risk in our cohort. Direct infection of hepatocytes and cholangiocytes has been proposed [17, 18]. However, single-cell RNA sequencing has indicated the sparse hepatocyte expression of receptors necessary for viral uptake. Hence, hepatocyte injury caused by SARS-CoV-2 may be more related to cytokine overproduction. The resulting systemic inflammatory response syndrome is linked to the lung-liver axis, leading to organ dysfunction [19, 20]. Inflammatory cytokine storms during COVID-19 infections are not uncommon and can result in sudden patient clinical deterioration and multiorgan failure [21]. Hence, decompensated liver disease appears to be a significant risk factor for mortality in patients with COVID-19. In addition, the cirrhotic liver has shown a more than a 30-fold increase in ACE2 receptor expression compared to healthy livers [22]. This finding highlights that cirrhotic patients may be uniquely susceptible to SARS-CoV-2-mediated hepatic dysfunction [20]. Furthermore, a study by Wanner et al. has shown that specific SARS-CoV-2 hepatotropic, further associating the ability of the virus to trigger decompensation in patients with pre-existing CLD [3, 23]. Patients with decompensated cirrhosis should be monitored closely to manage their disease-defining events and take extra precautions to minimize the risk of SARS-CoV-2 exposure.

Currently, the reason for the worse prognosis of COVID-19 patients with ALD remains unclear. Patients with ALD were notably more likely to require mechanical ventilation in our COVID-19 study cohort. A study by Wang et al. highlighted that the risk of severe COVID-19 was significantly associated with alcoholic liver damage (OR, 7.05; 95% CI, 6.30 to 7.88) and alcoholic liver cirrhosis (OR, 7.00; 95% CI, 6.15 to 7.97) [24]. ALD is associated with immune system dysregulation, leading to an increased risk of infection-related morbidity and mortality [25]. Moreover, ALD can suppress chemokine production and impair the expression of proteins that allow neutrophils to adhere to other cells at the site of infection [26]. Finally, ALD primes the alveolar epithelium for injury by promoting oxidative stress, increasing epithelial permeability and protein leak, and impairing fluid clearance through tight junction proteins within the epithelial barrier alterations [27]. These phenomena explain ALD patients' pathophysiological propensity to develop acute respiratory distress syndrome. ALD is associated with suppressing complement activation and systemic production of proinflammatory cytokines by various immune cells [28]. A direct effect of alcohol and alcohol-related effects on alveolar epithelial dysfunction and decreased concentration of pulmonary antioxidants in addition to immune function in individuals with chronic alcohol abuse [29]. In addition, patients with alcohol use disorder often have other comorbidities, such as smoking, metabolic syndrome, and chronic kidney disease, which have been independently associated with severe outcomes in SARS-CoV-2 patients [30, 31]. Patients with ALD had a 2.7-times higher hazard of mechanical ventilation in our cohort. Based on our findings, we could hypothesize that ALD patients with advanced stages are characterized by a severe condition, which negatively impacts their prognosis. In addition, a superimposed cytokine storm triggered by SARS-CoV-2 could exacerbate the heightened inflammatory state in patients with ALD, thus leading to worse outcomes.

Studies have shown that patients with abnormal liver chemistries have a significantly higher risk of developing severe pneumonia [20, 21, 32]. Our study findings suggest that elevated liver chemistries should be regarded as a red flag indicating a more severe disease course and major in-hospital outcomes in COVID-19 patients. Abnormal liver function occurs in the setting of direct hepatocyte injury caused by the SARS-CoV-2 and may be closely related to systemic inflammatory response syndrome. Our data imply a potential association between liver injury and the inflammatory responses induced by SARS-CoV-2 infection [21]. Given the profound multisystemic involvement in severe COVID-19, liver injury is likely to be multifactorial [22, 23]. Candidate mechanisms of liver injury include hypoxic hepatitis, drug-induced liver injury, intrahepatic immune activation, and microvascular thrombosis.

Our study has some limitations. First, it is retrospective and limited to a single healthcare system. The analysis represented only hospitalized patients, who were more likely to have severe COVID-19. The relationships detected in our study could be affected by collider bias due to the specific qualities of those hospitalized vs. those with the outcomes of interest [3]. In addition, there may have been referral bias due to the tertiary hospital setting of the study. However, our cohort represented an ethnically diverse population with varying stages of liver disease. We could also have enrollment bias because not all patients with CLD have documented ICD codes in their electronic health records. Despite our best attempts, we may not have identified all hospitalized patients with CLD and COVID-19. Although we attempted to collect data on the most relevant covariables, there remained a possibility of unmeasured confounding not captured by our registry, which was designed to allow rapid data entry during the pandemic's peak. Importantly, we had a smaller ALD and compensated cirrhosis sample due to undercoding and a lack of specific ICD-9-CM and ICD-10-CM codes. The causes of death in COVID-19 patients may involve multiple organ injuries, and it was challenging to differentiate CLD as the primary or direct cause of death. Our data were not able to include COVID-19 vaccines nor SARS-CoV-2 variants to assess the impact in accuracy in patients with CLD. We did not have data regarding treatment for autoimmune liver disease or viral hepatitis. Finally, we could not obtain long-term outcomes due to a comparatively short observation period. Further studies with long-term periods are needed to understand the long-term impact of COVID-19 on the liver and elucidate the pathogenic mechanisms.

## 5. Conclusion

Our study highlighted the myriad risk factors for poor clinical outcomes among patients with CLD and COVID-19 infection. Hepatic decompensation was associated with all-cause mortality, whereas ALD was independently associated with mechanical ventilation. In addition, liver enzymatic abnormalities may indicate more severe COVID-19 and help support clinical decisions regarding monitoring or risk stratification. Overall, our findings emphasized the need for patients with CLD to follow recommended preventive measures against SARS-CoV-2 exposure.

## Figures and Tables

**Table 1:** Baseline, clinical characteristics, and outcomes of patient with chronic liver disease and a positive test for SARS-CoV-2.

	All patients (*N* = 2,731)	Severity of COVID-19			Mortality status		
		Nonsevere^†^ (*N* = 544)	Severe^†^ (*N* = 2,187)	*P* value	Survivor (*N* = 2487)	Nonsurvivor (*N* = 244)	*P* value
*Age in years*, median (IQR)	61.3 (45.4–74.1)	52.4 (37.3–69.5)	62.6 (48.8–75.1)	<0.001	52.4 (37.3–69.5)	62.6 (48.8–75.1)	<0.001
*Sex, n (%)*							
Female	1403 (51.4)	296 (54.4)	1107 (50.6)	0.11	1289 (51.8)	114 (46.7)	0.13
*Ethnicity, n (%)* hispanic	680 (24.9)	154 (28.3)	526 (24.0)	0.04	649 (26.1)	31 (12.7)	<0.001

*Race, n (%)*							
White	936 (34.3)	158 (29.0)	778 (35.6)	0.002	824 (33.1)	112 (45.9)	<0.001
Black	884 (32.3)	192 (35.3)	692 (31.6)		808 (32.5)	76 (31.1)	
Asian	161 (5.9)	23 (4.2)	138 (6.3)		141 (5.7)	20 (8.2)	
Other	728 (26.6)	168 (30.9)	560 (25.6)		694 (27.9)	34 (13.9)	

*BMI (kg/m * ^*2*^ *), n (%)*							
≤18.5	70 (2.6)	15 (2.7)	55 (2.5)	<0.001	56 (2.2)	14 (5.70)	<0.001
18.5–24.9	565 (20.6)	125 (23.0)	440 (20.1)		489 (19.7)	76 (31.1)	
25–29.9	759 (27.8)	181 (38.3)	578 (26.4)		698 (28.1)	61 (25.0)	
≥30.0	1057 (38.7)	162 (29.8)	895 (40.9)		1007 (40.5)	50 (20.5)	

*Smoking status*							
Never	1731 (63.4)	362 (66.5)	1369 (62.6)	0.01	1621 (65.2)	110 (45.1)	<0.001
Former	565 (20.7)	92 (16.9)	473 (21.6)		496 (19.9)	69 (28.3)	
Current	267 (9.8)	66 (12.1)	201 (9.2)		245 (9.8)	22 (9.0)	

*Liver-related factors, etiology, n (%)*							
ALD	36 (1.3)	15 (2.7)	21 (1)	0.001	32 (1.3)	4 (1.6)	0.64
NAFLD	147 (5.4)	27 (5.0)	120 (5.5)	0.63	141 (5.7)	6 (2.4)	0.03
Viral hepatitis	480 (17.6)	108 (1989)	372 (17)	0.12	451 (18.1)	29 (11.9)	0.01
Other liver diseases	2548 (93.3)	502 (92.3)	2046 (93.6)	0.29	2314 (93.0)	234 (95.9)	0.08

*Cirrhosis*							
No cirrhosis	2436 (89.2)	484 (89.0)	1952 (89.3)	0.97	2227 (89.5)	209 (85.6)	0.16
CC	28 (1.0)	6 (1.1)	22 (1)		24 (1.0)	4 (1.6)	
DC	267 (9.8)	54 (9.9)	213 (9.7)		236 (9.5)	31 (12.7)	
HCC	9 (0.3)	2 (0.4)	7 (0.3)	0.86	5 (0.2)	4 (1.6)	<0.001

*Comorbidities, n (%)*							
*Cardiovascular disease*:							
CHF	521 (19.1)	48 (8.8)	473 (21.6)	<0.001	435 (17.5)	86 (35.2)	<0.001
HT without complications	1669 (61.1)	289 (53.1)	1380 (63.1)	<0.001	1486 (59.7)	183 (75.0)	<0.001
HT with complications	770 (28.2)	109 (20.0)	661 (30.2)	<0.001	659 (26.5)	111 (45.5)	<0.001

*Diabetes*:							
Diabetes without complications	935 (34.2)	145 (26.6)	790 (36.1)	<0.001	843 (33.9)	92 (37.7)	0.23
Diabetes with complications	793 (29.0)	98 (18.0)	695 (31.8)	<0.001	699 (28.1)	94 (38.5)	<0.001
Chronic respiratory disease	749 (27.4)	119 (21.9)	630 (28.8)	0.001	662 (26.6)	87 (35.6)	0.003
Chronic neurological disease	688 (25.2)	134 (24.6)	554 (25.3)	0.74	577 (23.2)	111 (45.5)	<0.001
CKD of any stage	490 (17.9)	72 (13.2)	418 (19.1)	0.001	411 (16.5)	79 (32.4)	<0.001
Anemia	1088 (39.8)	195 (35.8)	893 (40.8)	0.03	939 (37.7)	149 (61.1)	<0.001
HIV	48 (1.7)	12 (2.2)	36 (1.6)	0.37	46 (1.8)	2 (0.8)	0.24
Depression	698 (25.5)	146 (26.8)	552 (25.2)	0.44	613 (24.6)	85 (34.8)	<0.001
Hypothyroidism	373 (13.6)	56 (10.3)	317 (14.5)	0.01	317 (12.7)	56 (22.9)	<0.001

*Malignancies*:							
Primary cancer	285 (10.4)	46 (8.4)	239 (10.9)	0.09	241 (9.7)	44 (18.0)	<0.001
Metastatic cancer	182 (6.7)	28 (5.1)	154 (7.0)	0.11	152 (6.1)	30 (12.3)	<0.001

*Liver biochemistries*:							
*ALT,* median (IQR)	29(19–48)	24 (16–42)	30 (19–49)	<0.001	29 (19–49)	27 (17.5–45)	0.20
Normal, *n* (%)	784 (28.7)	222 (42.4)	562 (25.7)	<0.001	713 (28.7)	71 (29.1)	<0.001
1–2 ULN, *n* (%)	867 (31.7)	183 (34.9)	684 (31.3)		793 (31.9)	74 (30.3)	
>2–5 ULN, n (%)	729 (26.7)	82 (15.6)	647 (29.6)		684 (27.5)	45 (18.4)	
>5 ULN, *n* (%)	314 (11.5)	37 (7.1)	277 (12.7)		262 (10.7)	52 (21.3)	
*AST*, median (IQR)	36(25–55)	28 (21–43)	38 (27–57)	<0.001	35 (25–54)	42 (30–69)	<0.001
Normal, *n* (%)	1468 (53.7)	359 (66.0)	1109 (50.1)	<0.001	1363 (54.8)	105 (43.0)	<0.001
1–2 ULN, *n* (%)	839 (30.7)	99 (18.2)	740 (33.8)		754 (30.3)	85 (34.8	
>2–5 ULN, *n* (%)	240 (8.8)	36 (6.6)	204 (9.3)		209 (8.4)	31 (12.7)	
>5 ULN, *n* (%)	52 (1.9)	9 (1.6)	43 (2.0)		40 (1.6)	12 (4.9)	
*T. Bil*, median (IQR)	0.5 (0.3–0.6)	0.5 (0.3–0.6)	0.5 (0.3–0.6)	0.35	0.5 (0.3–0.6)	0.5 (0.4–0.9)	<0.001
Normal, *n* (%)	1468 (56.5)	359 (66.0)	1109 (50.7)	<0.001	2265 (91.1)	208 (85.2)	<0.001
1–2 ULN, *n* (%)	839 (30.7)	99 (18.2)	740 (33.8)		99 (4.0)	26 (10.6)	
>2–5 ULN, *n* (%)	240 (8.8)	36 (6.6)	204 (9.3)		19 (0.8)	4 (1.6)	
>5 ULN, *n* (%)	52 (1.9)	9 (1.6)	43 (2.0)		3 (0.1)	2 (0.8)	
*ALP*, median (IQR)	78 (61–103)	80 (65–108)	77 (61–101)	0.007	77 (61–101)	89(65–123)	<0.001
Normal, *n* (%)	2241 (82.0)	420 (77.2)	1821 (83.3)	0.22	2062 (82.9)	179 (73.4)	<0.001
1–2 ULN, *n* (%)	377 (13.8)	88 (16.2)	289 (13.2)		326 (13.1)	51 (20.9)	
>2–5 ULN, *n* (%)	54 (2.0)	11 (2.0)	43 (2.0)		43 (1.7)	11 (4.5)	
>5 ULN, *n* (%)	5 (0.2)	1 (0.2)	4 (0.2)		3 (0.1)	2 (0.8)	
GGT, median (IQR)	117(55–188)	119 (116–216)	115 (55–170)	0.47	117 (55–188)	116.5 (63–170)	0.93

*Clinical outcome, no. (%)*							
Still admitted	75 (2.7)	11 (2.0)	64 (2.9)	0.25	75 (3.0)	0 (0.0)	0.006
Discharged alive from the hospital	2412 (88.8)	533 (98.0)	1879 (85.9)	<0.001	2412 (97.0)	0 (0.0)	<0.001
Median length of hospital stay (IQR)	5.7 (3.0–10.2)	2.7 (1.8–4.4)	6.4 (3.8–11.8)	<0.001	5.4 (3.0–9.8)	8.2 (3.6–16.5)	<0.001
Died in the hospital	244 (8.9)	0 (0)	244 (11.1)	<0.001	0 (0.0)	244 (100)	<0.001

Abbreviations: IQR, interquartile range; BMI, body mass index; HT: hypertension; CHF, congestive heart failure; CKD, chronic kidney disease; CC, compensated cirrhosis; DC, decompensated cirrhosis; NAFLD, nonalcoholic fatty liver disease; ALD, alcohol-related liver disease; ALT, alanine aminotransferases; ALP, alkaline phosphatase; GGT: *γ*-glutamyl transpeptidase; AST: aspartate aminotransferase; T-Bil, total bilirubin; INR, international normalized ratio; BUN, blood urea nitrogen; PT, prothrombin time; CRP: C-reactive protein; LDH: lactate dehydrogenase. ^†^Severity of COVID-19 was graded as per the World Health Organization (WHO) interim guidance.

**Table 2:** Multivariable^∗^ Cox proportional hazards model for major outcomes among hospitalized patients with chronic liver disease and a positive test for SARS-CoV-2.

Clinical predictors	All-cause of mortality		Mechanical ventilation		Vasopressors	
	Multivariable HR (95% CI)	*P* value	Multivariable HR (95% CI)	*P* value	Multivariable HR (95% CI)	*P* value
*Age in years*	1.05 (1.04–1.07)	<0.001	1.00 (1.00–1.06)	0.03	0.99 (0.98–1.01)	0.35

*Sex*						
Female	Reference	0.45	Reference	0.43	Reference	0.47
Male	1.13 (0.82–1.55)		1.11 (0.86–1.43)		1.10 (0.84–1.42)	

*Etiology*						
ALD	2.75 (0.64–11.85)	0.17	2.79 (1.00–8.02)	0.05	2.27 (0.67–7.61)	0.18
NAFLD	0.21 (0.07–0.65)	0.006	0.54 (0.31–0.93)	0.02	0.66 (0.39–1.14)	0.14
Viral hepatitis	0.22 (0.09–0.50)	<0.001	0.49 (0.32–0.74)	0.001	0.56 (0.37–0.85)	0.007
Other liver diseases	0.36 (0.08–1.56)	0.17	0.36 (0.12–1.03)	0.06	0.44 (0.13–1.48)	0.19

*Cirrhosis*						
No cirrhosis	Reference		Reference		Reference	
CC	1.01 (0.13–8.15)	0.99	0.47 (0.10–2.17)	0.32	0.45 (0.10–2.08)	0.31
DC	2.94 (1.23–7.06)	0.02	1.51 (0.96–2.33)	0.07	1.45 (0.93–2.29)	0.11

*Comorbidities*						
CHF	1.13 (0.80–1.60)	0.48	1.75 (0.56–1.00)	0.05	0.78 (0.58–1.06)	0.11
HT	0.75 (0.48–1.17)	0.21	0.80 (0.56–1.13)	0.21	0.83 (0.57–1.21)	0.34
CKD	1.26 (0.87–1.82)	0.22	1.03 (0.77–1.38)	0.83	1.00 (0.74–1.35)	0.98
Diabetes	0.92 (0.65–1.31)	0.64	1.29 (0.98–1.70)	0.07	1.29 (0.96–1.73)	0.09
Chronic pulmonary disease	1.29 (0.92–1.83)	0.13	1.22 (0.93–1.62)	0.15	1.42 (1.07–1.91)	0.01
Chronic neurological disease	1.22 (0.88–1.71)	0.23	0.81 (0.60–1.08)	0.15	0.76 (0.56–1.03)	0.07
Primary malignancy	0.91 (0.62–1.33)	0.61	0.98 (0.68–1.42)	0.93	0.89 (0.61–1.32)	0.56
Metastatic malignancy	1.28 (0.84–1.96)	0.26	0.81 (0.51–1.27)	0.35	1.35 (1.01–1.59)	0.04
ALT	1.00 (1.00–1.00)	0.69	1.00 (0.99–1.00)	0.83	1.00 (1.00–1.00)	0.57
AST	1.00 (1.00–1.00)	0.43	1.00 (1.00–1.00)	0.53	1.00 (1.00–1.00)	0.85
ALP	1.02 (1.00–1.04)	<0.001	1.00 (1.00–1.01)	0.04	1.00 (1.00–1.01)	<0.001
T. Bil	1.25 (1.15–1.37)	<0.001	0.95 (0.76–1.18)	0.64	1.04 (0.89–1.20)	0.64
PT	1.04 (1.01–1.07)	0.007	0.02 (0.99–1.05)	0.19	1.03 (1.00–1.06)	0.07
INR	1.26 (1.01–1.57)	0.04	1.11 (0.88–1.39)	0.39	1.13 (1.09–1.42)	0.29
Neutrophil	1.00(1.00–1.00)	0.007	1.00 (1.00–1.00)	0.006	1.00 (1.00–1.00)	0.002
BUN	1.01 (1.01–1.02)	0.003	1.01 (1.00–1.02)	0.03	1.01 (1.00–1.02)	0.003
CRP	1.05 (1.01–1.14)	0.008	1.00 (1.00–1.00)	0.21	1.00 (1.00–1.00)	0.42
D-Dimer	1.04 (1.01–1.07)	0.02	0.98 (0.95–1.01)	0.27	1.01 (0.98–1.04)	0.47
LDH	1.00 (1.00–1.01)	0.002	1.00 (1.00–1.00)	0.05	1.00 (1.00–1.00)	0.10
Ferritin	1.00 (0.98–1.00)	0.14	1.00 (1.00–1.00)	0.03	1.00 (1.00–1.00)	0.09

*ALT*						
Normal,	Reference		Reference		Reference	
1-2 ULN	0.86 (0.57–1.30)	0.48	1.48 (0.95–2.30)	0.08	1.34 (0.84–2.14)	0.22
>2–5 ULN	1.47 (1.29–2.77)	0.003	1.11 (0.73–1.70)	0.62	1.04 (0.66–1.63)	0.87
>5 ULN	1.21 (0.74–1.99)	0.45	1.16 (0.73–1.84)	0.54	1.14 (0.70–1.85)	0.59

*AST*						
Normal,	Reference		Reference		Reference	
1-2 ULN	0.94 (0.67–1.33)	0.74	1.17 (0.89–1.53)	0.26	1.32 (1.00–1.76)	0.05
>2–5 ULN	1.92 (1.10–3.34)	0.02	1.69 (1.09–2.61)	0.01	1.55 (0.97–2.50)	0.07
>5 ULN	3.62 (1.52–8.64)	0.004	2.50 (1.12–5.58)	0.02	2.81 (1.18–6.71)	0.02

*Bilirubin*						
Normal	Reference		Reference		Reference	
1-2 ULN	1.24 (0.70–2.23)	0.45	1.19 (0.73–1.95)	0.48	1.49 (0.93–2.39)	0.09
>2–5 ULN	2.11 (0.49–9.03)	0.31	0.69 (0.16–2.97)	0.62	0.85 (0.20–3.67)	0.83
>5 ULN	5.21 (0.66–40.91)	0.12	0.00 (IO)	NS	0.00 (IO)	N0

*ALP*						
Normal	Reference		Reference		Reference	
1-2 ULN	1.68 (1.12–2.52)	0.01	1.17 (0.84–1.65)	0.36	1.28 (0.90–1.84)	0.17
>2–5 ULN	2.29 (1.10–5.27)	0.05	1.03 (0.41–2.59)	0.94	1.80 (0.81–3.98)	0.15
>5 ULN	2.15 (0.44–10.43)	0.34	0.57 (0.08–4.22)	0.58	1.44 (0.34–6.12)	0.62

Abbreviations: HT, hypertension; CHF, congestive heart failure; CKD, chronic kidney disease; CC, compensated cirrhosis; DC, decompensated cirrhosis; NAFLD, nonalcoholic fatty liver disease; ALD, alcohol-related liver disease; ALT, alanine aminotransferases; ALP, alkaline phosphatase; GGT: *γ*-glutamyl transpeptidase; AST, aspartate aminotransferase; T-Bil, total bilirubin; INR, international normalized ratio; BUN, blood urea nitrogen; PT, prothrombin time; CRP, C-reactive protein; LDH, lactate dehydrogenase; HR, hazard ratio; CI, confidence interval, IO, insufficient observation. ^∗^Age, gender, ethnicity, race, body mass index, smoking use, etiology of liver diseases, and all the pre-existing comorbidities were adjusted as confounders in the multivariable Cox proportional hazards model.

## Data Availability

There are no additional data available.

## Abbreviations

COVID-19: Coronavirus disease 2019
SARS-CoV-2: Severe acute respiratory syndrome coronavirus-2
CLD: Chronic liver diseases
MV: Mechanical ventilation
LD: Liver diseases
ALD: Alcoholic related liver disease
HR: Hazard ratios
CI: Confidence intervals
IQR: Interquartile range
HT: Hypertension
DM: Diabetes mellitus
COPD: Chronic obstructive pulmonary disease
BUN: Blood urea nitrogen
PT: Prothrombin time
INR: International normalized ratio
ULN: Upper limit of the normal-range
AST: Aspartate aminotransferase
T. Bil: Total bilirubin
ALP: Alkaline phosphatase
GGT: Gamma-glutamyl transpeptidase
ALT: Alanine aminotransferase
CRP: C-reactive protein
NAFLD: Nonalcoholic fatty liver disease
BMI: Body mass index.

## Supplementary Materials

Supplementary Materials Supplementary materials provided details of methodology describing the clinical classification and definitions of the study cohort. Supplementary Table 1: ICD-10 codes indicating etiologies of liver diseases. Supplementary Table 2: laboratory findings and in-hospital management of patients with chronic liver disease and a positive test for SARS-CoV-2. Supplementary Table 3: clinical characteristics of patient's major outcome as the need for mechanical ventilation and vasopressor support for patients with chronic liver disease and a positive test for SARS-CoV-2.
