# Supplementary material for: Risk of Severe Illness and Risk Factors of Outcomes of COVID-19 in Hospitalized Patients with Chronic Liver Disease in a Major U. S. Hospital Network
Source: Can J Gastroenterol Hepatol. 2022 Nov 3;2022:8407990. doi: 10.1155/2022/8407990 (PMC9649328; doi:10.1155/2022/8407990)
Supplement: Supplementary Materials — provided details of methodology describing the clinical classification and definitions of the study cohort. Supplementary Table 1: ICD-10 codes indicating etiologies of liver diseases. Supplementary Table 2: laboratory findings and in-hospital management of patients with chronic liver disease and a positive test for SARS-CoV-2. Supplementary Table 3: clinical characteristics of patient's major outcome as the need for mechanical ventilation and vasopressor support for patients with chronic liver disease and a positive test for SARS-CoV-2. [file 8407990.f1.doc]

**SUPPLEMENTARY MATERIAL:**

**Clinical Classification:**

The severity of COVID-19 was graded as per the World Health Organization (WHO) interim guidance.[[1]](#endnote-2) Asymptomatic patients or those with only mild upper respiratory tract symptoms and without abnormalities on chest imaging were defined as having a mild disease. Those who had respiratory tract symptoms with no obvious hypoxemia and pneumonia manifestation by imaging were defined as having moderate. Patients having resting fingertip oxygen saturation <90% on room air or a respiratory rate ≥30 breaths/minute; a ratio of the arterial partial pressure of oxygen to fraction of inspired oxygen (PaO2/FiO2) <300 mmHg or lung infiltrates >50% were defined as severe COVID-19. The critical disease was classified if the patient had respiratory failure requiring mechanical ventilation, or symptoms of shock, or respiratory failure combined with other organ dysfunction requiring intensive care. In addition, based on the above classification, identified COVID-19 patients were then stratified into two groups: non-severe (mild and moderate cases) or severe (severe and critical cases) disease.

**Definitions:**

An Upper limit of the normal range (ULN) of ALT was defined according to the criteria of the American Association for the Study of Liver Diseases (greater than 25 U/L for women and 35 U/L for men).[[2]](#endnote-3) In addition, abnormal levels of other liver chemistries were based on patients having an ULN of aspartate aminotransferase (AST), total bilirubin (T-Bil), alkaline phosphatase (ALP), and gamma-glutamyl transpeptidase (GGT). Furthermore, the degree of liver injury was grouped based on the degree of liver enzyme elevation as mild (1-2 times of ULN), moderate (>2-5 times of ULN), and severe (>5 times of ULN). CLD diagnoses were made based on an International Classification of Diseases, 10th Revision code (ICD-10-CM diagnosis codes) for liver diseases at any time before the index date. Diagnoses of cirrhosis were made based on previously validated ICD-10-CM codes for cirrhosis and its complications (Supplementary Table 1) at any time before the index date. Decompensation was defined as the presence of jaundice, variceal bleed, ascites, or hepatic encephalopathy at baseline and during COVID-19 were collected. We excluded the patients who had undergone orthotopic liver transplantation and had unclear diagnoses of CLD or COVID-19. Only the first admission was analyzed for those admitted multiple times during this period.

**Supplementary** **Table 1: ICD-10 codes indicating etiologies of liver diseases**

| **Etiology for liver diseases** | **ICD-10** |
| --- | --- |
| Alcoholic Liver Disease | K70.(0,9,10,30) |
| Hepatitis B virus | B16.x, B17.0, B18.(0,1), B19.1x, Z22. 51 |
| Hepatitis C virus | B17.1x, B18.2, B19.2x, Z22.52, Z86.19 |
| Hepatitis A virus | B15.x |
| Other viral hepatitis | B17.(2, 8,9),B18.(8,9), B19.x, K73.x, K71.6, |
| Carrier or suspected carrier of hepatitis B, hepatitis C, unspecified, and other viral hepatitis | Z22.5x |
| NAFLD/NASH/NASH-cirrhosis | K74.(0,1,2), K74.6x, K75.81, K76.(0,89, 9) |
| Biliary cirrhosis | K74.(3,4,5) |
| Budd-Chiari syndrome | I82.0 |
| Autoimmune hepatitis | K75.4 |
| Hemochromatosis/ iron overload(IO): | E83.11x |
| Other liver diseases | E75.2x,249, E77.x |
| Other specified/unspecified disorders of the liver | K71.xx, K74.4, K75.x, K76.(2-5) |
| Wilson's disease | E83.0x |
| Hepatocellular carcinoma | C22.(0,1,2,7,8) |
| Codes for compensated cirrhosis: | |
| Alcoholic cirrhosis of the liver without ascites | [K70.30](https://www.icd10data.com/ICD10CM/Codes/K00-K95/K70-K77/K70-/K70.30) |
| Unspecified/other cirrhosis of the liver | K74.(60,69) |
| Biliary cirrhosis | K74.(3,4,5) |
| Codes for cirrhosis related complications: | |
| Spontaneous bacterial peritonitis | K65.2 |
| Hepatic encephalopathy | K72.91 |
| Esophageal varices | I85.xx |
| Hepatopulmonary Syndrome | K76.81 |
| Hydrothorax | J90, J94.8 |
| Acute kidney injury | N17.9, N28.9 |
| Ascites | K70.(11,31), K71.51, R18.8 |
| Hepatorenal syndrome | K76.7 |

**Supplementary Table 2. Laboratory Findings and In-hospital management of patients With Chronic Liver Disease and a positive test for SARS-CoV-2**

| **Variables** | **All patients**  (N =**2,731**) | **Severity of COVID-19** | | | **Mortality status** | | |
| --- | --- | --- | --- | --- | --- | --- | --- |
|  |  | **Non-severe†**  (N =**544**) | **Severe†**  (N =**2187**) | **P-value** | **Survivor**  (N =**2487 )** | **Non-Survivor**  (N =**244**) | **P-value** |
| **Vital signs:** median (IQR) | | | | | | | |
| *Temperature (°C)*  *SBP (mm Hg)*  *DBP(mm Hg)*  *O^2^ saturation (%)*  *Respiratory rate/*  *per min*  *Fio2* | 37 (36.7-37.4)  125 (112 -140)  71 (62 -79)  96 (94 -98)  20 (18 -22)  40 (21-60) | 36.9 (36.7-37.3)  125 (114 -140)  72 (63-80)  97 (95.5-98.5)  18 (17-20)  21 (21-21) | 37.1 (36.7-37.5)  125 (112 -140)  70 (62 -79)  96 (94 -98)  20 (18 -22)  40 (21 -60) | <0.001  0.27  0.011  <0.001  <0.001  <0.001 | 37 (36.7-37.5)  125 (113 -139)  71 (63-80)  96 (94-98)  19 (18-22)  31 (21 -60) | 36.9 (36.6-37.3)  122 (106 -142)  68 (56 -76)  95 (92-97)  22(18 -26)  60 (40 -100) | <0.001  0.065  <0.001  <0.001  <0.001  <0.001 |
| **Coagulation test,** median (IQR) | | | | | | | |
| *PT (sec)* | 11 (10.5-11.8) | 10.9 (10.3-11.6) | 11 (10.5-11.9) | <0.001 | 10.9 (10.4-11.7) | 11.7 (10.9-13.1) | <0.001 |
| *INR* | 1.1 (1 -1.1) | 1.05 (1 -1.1) | 1.1 (1 -1.1) | 0.010 | 1.1 (1 -1.1) | 1.1 (1.05-1.3) | <0.001 |
| *APTT (sec)* | 26.3 (1.2-31.7) | 25.8 (1.1-29.8) | 26.5 (1.2-32) | 0.039 | 26.4 (1.2-31.5) | 26 (1.6-34) | 0.025 |
| *D-Dimer* | 0.9 (0.5-1.8) | 0.9 (0.5-1.8) | 0.9 (0.5-1.8) | 0.65 | 0.83 (0.50-1.64) | 1.83 (1.12-3.61) | <0.001 |
| **Routine blood tests,** median (IQR) | | | | | | | |
| *Hemoglobin (g/dL)* | 12.1 (10.4-13.5) | 12.3 (10.7-13.6) | 12.1 (10.3-13.4) | 0.075 | 12.2 (10.6-13.5) | 10.5 (8.8-12.6) | <0.001 |
| *WBC (/mcL)* | 7.1 (5.1-10.03) | 6.4 (4.6-9.3) | 7.24 (5.2-10.2) | <0.001 | 6.9 (5-6) | 10.02 (6.8-15.6) | <0.001 |
| *RBC (/mcL)* | 4.1 (3.5-4.7) | 4.2 (3.5-4.7) | 4.13 (3.5-4.7) | 0.58 | 4.16 (3.54-4.71) | 3.66 (3.04-4.48) | <0.001 |
| *Platelets (/mcL)* | 231 (173 -308) | 216 (166 -287) | 236 (176 -313) | <0.001 | 233 (177 -311) | 213.5 (146.5-269.5) | <0.001 |
| *Neutrophils(/mcL)* | 14.9 (4.9-71.6) | 14.3 (4.2-66.3) | 15.6 (5.1-72.8) | <0.001 | 14.11 (4.7-70.8) | 40.2 (6.9-80.3 | <0.001 |
| *Lymphocytes (/mcL)* | 16.9 (10.5-25.2) | 21.4 (14 -30) | 16.00 (9.9-23.9) | <0.001 | 17.8 (11.1-26) | 9.00 (5 -13.9) | <0.001 |
| **Renal function tests,** median (IQR) | | | | | | | |
| *Creatinine (mg/dL)* | 0.8 (0.7-1.1) | 0.8 (0.6-1) | 0.8 (0.7-1.1) | 0.023 | 0.8 (0.7-1) | 1.01 (0.7-1.8) | <0.001 |
| *BUN, (mmol/L)* | 15 (10 -23) | 11 (8 -16) | 16 (1 -24) | <0.001 | 14 (10 -21) | 27 (19 -42) | <0.001 |
| *Sodium (mEq/L)* | 138 (136 -140) | 138 (136 -140) | 138 (136 -141) | 0.010 | 138 (136 -140) | 139 (136 -144) | <0.001 |
| *Potassium (mEq/L)* | 4.10 (3.80-4.40) | 4 (3.7-4.3) | 4.1 (3.8-4.5) | <0.001 | 4.1 (3.8-4.4) | 4.1 (3.7-4.6) | 0.058 |
| *Total protein (g/L)* | 6.6 (6 -7.1) | 6.7 (6.2-7.2) | 6.5 (6 -7.1) | <0.001 | 6.6 (6.1-7.1) | 6.2 (5.5-6.9) | <0.001 |
| *Serum albumin, g/dL* | 3.8 (3.4-4.1) | 4 (3.5-4.3) | 3.8 (3.4-4.1) | <0.001 | 3.8 (3.4-4.1) | 3.6 (3-4) | <0.001 |
| **Inflammatory markers,** median (IQR) | | | | | | | |
| *IL-6 (pg/mL)* | 31.6 (12.3-78.7) | 17.3 (7.3-38.6) | 36.3 (14 -88.7) | <0.001 | 28.6 (11.4-68.1) | 101 (62.9-286) | <0.001 |
| *Ferritin (ng/mL)* | 553 (244 -1043) | 317.5 (108 -700) | 605(279 -1125) | <0.001 | 528 (235 -982) | 901.5 (352 -1677) | <0.001 |
| *CRP (mg/L)* | 7.9 (3.2-20.1) | 3.1 (1.2-7.4) | 9.6 (4.2-22.7) | <0.001 | 7.4 (3 -17.6) | 21.3 (7.5-83.3) | <0.001 |
| *Fibrinogen* (*mg/dL)* | 489 (387 -611) | 423 (337 -549) | 506(396-622) | <0.001 | 495 (389 -612) | 472 (300 -603) | 0.032 |
| *Lactate(mmol/L)* | 1.4 (1 -1.9) | 1.2 (1 -1.6) | 1.4 (1 -1.9) | 0.022 | 1.3 (1 -1.8) | 1.9 (1.3-2.6) | <0.001 |
| *ESR (mm/hr)* | 50 (29 -72) | 41(24 -57) | 52 (31-75) | <0.001 | 50 (29 -72) | 50.5 (27.5-74.5) | 0.97 |
| **Cardiac markers,** median (IQR) | | | | | | | |
| *Troponin I* *(ng/L)* | 0.1 (0.04-0.2) | 0.05 (0.03-0.1) | 0.1 (0.04-0.2) | <0.001 | 0.1 (0.04-0.2) | 0.13 (0.05-0.4) | <0.001 |
| *LDH (U/L)* | 320 (239 -433.5) | 260 (193 -345) | 336 (253 -456) | <0.001 | 315 (236 -420) | 443 (299 -624) | <0.001 |
| **Pharmacological treatment,** n(%) | | | | | | | |
| *NSAIDs* | 2382 (87.2) | 465 (85.5) | 1917 (87.7) | 0.17 | 2180 (87.7) | 202 (82.8) | 0.030 |
| *Antiviral therapy* | 128 (4.7) | 16 (2.9) | 112(5.1) | 0.031 | 117 (4.7) | 11 (4.5) | 0.89 |
| *Antibacterial therapy* | 1803 (66) | 309 (56.8) | 1494 (68.3) | <0.001 | 1574 (63.3) | 229 (93.9) | <0.001 |
| *Antifungal therapy* | 121 (4.4) | 11 (2) | 110 (5) | 0.002 | 90 (3.6) | 31 (12.7) | <0.001 |
| *Azithromycin* | 697 (25.5) | 96 (17.6) | 601 (27.5) | <0.001 | 616 (24.8) | 81 (33.2) | 0.004 |
| *Hydroxychloroquine* | 321 (11.8) | 36 (6.6) | 285 (13) | <0.001 | 280 (11.3) | 41 (16.8) | 0.010 |
| *Oseltamivir* | 8 (0.3) | 1 (0.2) | 7 (0.3) | 0.60 | 7 (0.3) | 1 (0.4) | 0.72 |
| *Remdesivir* | 49 (1.8) | 0 (0.0) | 49 (2.2) | <0.001 | 812 (32.6) | 62 (25.4) | 0.021 |
| *Vitamin D* | 251 (9.2) | 42 (7.7) | 209 (9.6) | 0.18 | 227 (9.1) | 24 (9.8) | 0.71 |
| *Statins* | 917 (33.6) | 133 (24.4) | 784 (35.8) | <0.001 | 827 (33.3) | 90 (36.9) | 0.25 |
| *ACE inhibitors* | 259 (9.5) | 51 (9.4) | 208 (9.5) | 0.92 | 243 (9.8) | 16 (6.6) | 0.10 |
| *ARB inhibitors* | 272 (10) | 45 (8.3) | 227 (10.4) | 0.14 | 253 (10.2) | 19 (7.8) | 0.23 |
| **Immunomodulatory therapy, no. (%)** | | | | | | | |
| *Dexamethasone* | 1019(37.3) | 65 (11.9) | 954 (43.6) | <0.001 | 932 (37.5) | 87 (35.7) | 0.58 |
| *Tocilizumab* | 49 (1.8) | 0(0) | 49 (2.2) | <0.001 | 37 (1.5) | 12 (4.9) | <0.001 |
| **Advanced therapies, no. (%)** | | | | | | | |
| *Vasopressors* | 307 (11.2) | 0 (0) | 307 (14) | <0.001 | 200 (8) | 107 (43.9) | <0.001 |
| *RRT/Dialysis* | 140 (5.1) | 1 (0.2) | 139 (6.4) | <0.001 | 123 (4.9) | 17 (7) | 0.17 |
| **Abbreviations:** IQR, interquartile range; O, oxygen; SBP, systolic blood pressure; DBP, diastolic blood pressure; Fio2, fraction of inspired  Oxygen; RBC, red blood cells; WBC, white blood cell; BUN, blood urea nitrogen; IL,interleukin; ESR, erythrocyte sedimentation rate; PT- prothrombin time; INR- international normalized ratio; APTT- activated partial thromboplastin time; LDH, lactate dehydrogenase; CRP, C reactive protein; RRT,renal replacement therapy.  †- Based on the World Health Organization disease severity classification | | | | | | | |

**Supplementary Table 3:** **Clinical characteristics of patient Major outcome as the need for mechanical ventilation and Vasopressor support of patients with Chronic Liver Disease and a positive test for SARS-CoV-2**

| **Variables** | **All patients**  (N =**2,731**) | **No Mechanical ventilation**  **(n=2381 )** | **Mechanical ventilation**  **(n=350)** | **P-value** | **No Vasopressor support**  **(n=2424)** | **Vasopressor support**  **(n=307)** | **P-value** |
| --- | --- | --- | --- | --- | --- | --- | --- |
| **Age in years,**  median (IQR) | 61.3  (45.4-74.1) | 60.8  (44.7-74.3) | 64  (51.5-73.1) | 0.032 | 60.6  (44.2-74.2) | 65.5  (54.4-73.5) | <0.001 |
| **Sex,** *Female* | 1403 (51.4) | 1266 (53.2) | 137 (39.1) | <0.001 | 1,283 (52.9) | 120 (39.1) | <0.001 |
| **Ethnicity,** *Hispanic* | 680 (25.1) | 594 (25.1) | 86 (24.7) | 0.87 | 611 (25.4) | 69 (22.6) | 0.29 |
| **Race,** n (%)  *White*  *African American*  *Asian*  *Other* | 936 (34.6)  884 (32.6)  161 (5.9)  728 (26.9) | 824 (34.9)  764 (32.3)  140 ( 5.9)  635 (26.9) | 112 (32.4)  120 (34.7)  21 (6.1)  93 (26.9) | 0.78 | 840 (34.9)  777 (32.3)  139 (5.8)  650 (27) | 96 (31.7)  107 (35.3)  22 (7.3)  78 (25.7) | 0.44 |
| **BMI (kg/m2),** n (%)  *≤18.5*  *18.5 – 24.9*  *25–29.9*  *≥30.0* | 70 (2.9)  565 (23.1)  759 (31.0)  1057 (43.1) | 61 (2.8)  499 (23.3)  661 (30.9)  920 (43) | 9 (2.9)  66 (21.3)  98 (31.6)  137 (44.2) | 0.89 | 62 (2.9)  499 (23)  673 (31)  936 (43.1) | 8 (2.8)  66 (23.5)  86 (30.6)  121 (43.1) | 1.00 |
| **Liver-related factors,** n (%) | | | | | | | |
| **Etiology**  *ALD*  *NAFLD*  *Viral hepatitis*  *Other liver diseases* | 36 (1.3)  147 (5.4)  480 (17.6)  2548 (93.3) | 31 (1.3)  126 (5.3)  397 (16.7)  2224 (93.4) | 5 ( 4)  21 (6)  83 (23.7)  324 (92.6) | 0.85  0.58  0.001  0.56 | 31 (1.3)  125 (5.2)  400 (16.5)  2,268 (93.6) | 5 (1.6)  22 (7.2)  80 (26.1)  280 (91.2) | 0.61  0.14  <0.001  0.12 |
| **Cirrhosis**  *No cirrhosis*  *CC*  *DCC* | 2436 (89.2)  28 (1)  267 (9.8) | 2158 (90.6)  24 (1)  199 (8.4) | 278 (79.4)  4 (1.1)  68 (19.4) | <0.001 | 2199 (90.7)  24 (1)  201 (8.3) | 237 (77.2)  4 (1.3)  66 (21.5) | <0.001 |
| *HCC* | 9 ( 0.3) | 8 (0.3) | 1 (0.3) | 0.88 | 7 (0.3) | 2 (0.7) | 0.30 |
| **Comorbidities,** n (%) | | | | |  |  |  |
| ***Cardiovascular disease:***  *CHF*  *HT without complications*  *HT with complications* | 521 (19.1)  1669 (61.1)  770 (28.2) | 411 (17.3)  1,424 (59.8)  622 (26.1) | 110 (31.4)  245 (70)  148 (42.3) | <0.001  <0.001  <0.001 | 416 (17.2)  1447 (59.7)  635 (26.2) | 105 (34.2)  222 (72.3)  135 (44) | <0.001  <0.001  <0.001 |
| ***Diabetes:***  *Diabetes without complications*  *Diabetes with complications* | 935 (34.2)  793 (29) | 775 (32.5)  630 (26.5) | 160 (45.7)  163 (46.6) | <0.001  <0.001 | 787 (32.5)  644 (26.6) | 148 (48.2)  149 (48.5) | <0.001  <0.001 |
| *Chronic respiratory disease* | 749 (27.4) | 633 (26.6) | 116 (33.1) | 0.010 | 643 (26.5) | 106 (34.5) | <0.001 |
| *HIV* | 48 (1.8) | 39 (1.6) | 9 (2.6) | 0.21 | 39 (1.6) | 9(2.6) | 0.21 |
| *Depression* | 698 (25.6) | 591 (24.8) | 107 (30.6) | 0.021 | 603 (24.9) | 95 (30.9) | 0.022 |
| *Chronic neurological disease* | 688 (25.2) | 566 (23.8) | 122 (34.9) | <0.001 | 581 (24) | 107 (34.9) | <0.001 |
| *CKD of any stage* | 490 (17.9) | 383 (16.1) | 107 (30.6) | <0.001 | 388 (16) | 102 (33.2) | <0.001 |
| *Anemia* | 1088 (39.8) | 870 (36.5) | 218 (62.3) | <0.001 | 892 (36.8) | 196 (63.8) | <0.001 |
| ***Malignancies****:*  *Primary cancer*  *Metastatic cancer* | 285 (10.4)  182 (6.7) | 243 (10.2)  160 (6.7) | 42 (12)  22 (6.3) | 0.31  0.76 | 244 (10.1)  165 (6.8) | 41 (13.4)  17 (5.5) | 0.076  0.40 |
| **Liver biochemistries:** | | | | | | | |
| **ALT,** median (IQR) | 29(19 -48) | 28 (18-47) | 33(21 -55) | 0.004 | 28 (18-48) | 32 (21-55.5) | 0.001 |
| Normal, n(%) | 784 (29.1) | 742 (31.7) | 42 (12) | <0.001 | 748 (31.3) | 36 (11.7) | <0.001 |
| 1-2 ULN, n(%) | 867 (32.2) | 778 (33.2) | 89 (25.4) |  | 0 (0) | 0 (0) |  |
| >2-5 ULN, n(%) | 729 (27.1) | 601 (25.6) | 128 (36.6) |  | 615 (25.8) | 114 (37.1) |  |
| >5 ULN, n(%) | 314 (11.7) | 223 (9.5) | 91 (26) |  | 0 (0) | 0 (0) |  |
| **AST,** median (IQR) | 36(25 -55) | 35(25-53) | 43 (30-66) | <0.001 | 35 (25-54) | 43 (31-65) | <0.001 |
| Normal, n(%) | 1468 (56.5) | 1330 (58.8) | 138 (40.7) | <0.001 | 1346 (58.6) | 122 (40.5) | <0.001 |
| 1-2 ULN, n(%) | 839 (32.3) | 690 (30.5) | 149 (44) |  | 0 (0) | 0 (0) |  |
| >2-5 ULN, n(%) | 240 (9.2) | 203 (9) | 37 (10.9) |  | 208 (9.1) | 32 (10.6) |  |
| >5 ULN, n(%) | 52 (2) | 37 (1.6) | 15 (4.4) |  | 41 (1.8) | 11 (3.7) |  |
| **Bilirubin,** median (IQR) | 0.5 (0.3-0.6) | 0.5 (0.3-0.6) | 0.5 (0.3-0.7) | 0.003 | 0.5 (0.3-0.6) | 0.5 (0.4-0.8) | <0.001 |
| Normal, n(%) | 1468 (56.5) | 2166 (95) | 307 (88.7) | <0.001 | 2206 (95) | 267 (88.1) | <0.001 |
| 1-2 ULN, n(%) | 839 (32.3) | 91 (4) | 34 (9.8) |  | 93 (4) | 32 (10.6) |  |
| >2-5 ULN, n(%) | 240 (9.2) | 18 (0.8) | 5 (1.4) |  | 19 (0.8) | 4 (1.3) |  |
| >5 ULN, n(%) | 52 (2) | 5 (0.2) | 0 (0) |  | 5 (0.2) | 0 (0) |  |
| **ALP,** median (IQR) | 78 (61 -103) | 77 (61 -101) | 82 (63 -113) | 0.053 | 77 (61-101) | 80 (62 -114) | 0.17 |
| Normal, n(%) | 2,241 (83.7) | 1970 (84.5) | 271 (78.1) | 0.016 | 2002 (84.4) | 239 (78.4) | 0.014 |
| 1-2 ULN, n(%) | 377 (14.1) | 309 (13.3) | 68 (19.6) |  | 320 (13.5) | 57 (18.7) |  |
| >2-5 ULN, n(%) | 54 (2) | 47 (2) | 7 (2) |  | 47 (2) | 7 (2.3) |  |
| >5 ULN, n(%) | 5 (0.2) | 4 (0.2) | 1 (0.3) |  | 3 (0.1) | 2 (0.7) |  |
| **GGT,** median (IQR) | 117 (55 -188) | 117.5 (55 -199) | 117 (95-119) | 0.57 | 117.5 (55 -199) | 117 (95-119) | 0.57 |
| **Abbreviations:** IQR, interquartile range; BMI, body mass index; ALD, alcohol-related liver disease; NAFLD, nonalcoholic fatty liver disease; CC.compensated cirrhosis; DC, decompensated cirrhosis; HCC, hepatocellular carcinoma;HT , hypertension; HIV, human immunodeficiency virus; CHF, congestive heart failure; CKD, chronic kidney disease; ALT, alanine aminotransferases; AST, aspartate aminotransferase; ALP, alkaline phosphatase; GGT: γ-glutamyl transpeptidase; T-Bil, total bilirubin; ULN, upper limit of normal. | | | | | | | |

**Supplementary references:**

1. . WHO. Clinical management of severe acute respiratory infection when Novel coronavirus (nCoV) infection is suspected: interim guidance. 2020. [↑](#endnote-ref-2)
2. . Terrault NA, Lok ASF, McMahon BJ, et al. Update on prevention, diagnosis, and treatment of chronic hepatitis B: AASLD 2018 hepatitis B guidance. *Hepatology.* 2018;67(4):1560-1599. [↑](#endnote-ref-3)
